# Differential Brain and Muscle Tissue Oxygenation Responses to Exercise in Tibetans Compared to Han Chinese

**DOI:** 10.3389/fphys.2021.617954

**Published:** 2021-02-24

**Authors:** Jui-Lin Fan, Tian Yi Wu, Andrew T. Lovering, Liya Nan, Wang Liang Bang, Bengt Kayser

**Affiliations:** ^1^Department of Physiology, Faculty of Medical and Health Sciences, University of Auckland, Auckland, New Zealand; ^2^Research Center for High Altitude Medicine, Tibet University Medical College, Lhasa, China; ^3^National Key Laboratory of High Altitude Medicine, Xining, China; ^4^Department of Human Physiology, University of Oregon, Eugene, OR, United States; ^5^Institute of Sport Sciences, University of Lausanne, Lausanne, Switzerland

**Keywords:** Tibetans, exercise performance, hypoxia, muscle tissue oxygenation, cerebral tissue oxygenation

## Abstract

The Tibetans’ better aerobic exercise capacity at altitude remains ill-understood. We tested the hypothesis that Tibetans display better muscle and brain tissue oxygenation during exercise in hypoxia. Using near-infrared spectrometry (NIRS) to provide indices of tissue oxygenation, we measured oxy- and deoxy-hemoglobin ([O_2_Hb] and [HHb], respectively) responses of the vastus lateralis muscle and the right prefrontal cortex in ten Han Chinese and ten Tibetans during incremental cycling to exhaustion in a pressure-regulated chamber at simulated sea-level (air at 1 atm: normobaric normoxia) and 5,000 m (air at 0.5 atm: hypobaric hypoxia). Hypoxia reduced aerobic capacity by ∼22% in both groups (*d* = 0.8, *p* < 0.001 vs. normoxia), while Tibetans consistently outperformed their Han Chinese counterpart by ∼32% in normoxia and hypoxia (*d* = 1.0, *p* = 0.008). We found cerebral [O_2_Hb] was higher in Tibetans at normoxic maximal effort compared Han (*p* = 0.001), while muscle [O_2_Hb] was not different (*p* = 0.240). Hypoxic exercise lowered muscle [O_2_Hb] in Tibetans by a greater extent than in Han (interaction effect: *p* < 0.001 vs. normoxic exercise). Muscle [O_2_Hb] was lower in Tibetans when compared to Han during hypoxic exercise (*d* = 0.9, *p* = 0.003), but not during normoxic exercise (*d* = 0.4, *p* = 0.240). Muscle [HHb] was not different between the two groups during normoxic and hypoxic exercise (*p* = 0.778). Compared to Han, our findings revealed a higher brain tissue oxygenation in Tibetans during maximal exercise in normoxia, but lower muscle tissue oxygenation during exercise in hypoxia. This would suggest that the Tibetans privileged oxygenation of the brain at the expense of that of the muscle.

## Introduction

Aerobic performance is an important determinant of one’s ability to thrive at high altitude, yet the exact mechanisms limiting aerobic performance at high altitude remain poorly understood. Tibetans are renowned for their superior exercise performance at high altitude compared to their Western and Han Chinese counterparts (Wu et al., 2005; Marconi et al., 2006; Wu and Kayser, 2006; Gilbert-Kawai et al., 2014; Kayser et al., 2019). While this remarkable performance has been largely attributed to better systemic oxygen (O_2_) transport in Tibetans, it is unknown whether this translates into better muscle and/or brain tissue oxygenation during exercise.

Within the skeletal muscles, Tibetans and Sherpas display lower mitochondrial volume density and muscle fiber cross-sectional area, and higher muscle capillary density and myoglobin concentration compared to lowlanders (Kayser et al., 1991, 1996; Erzurum et al., 2007). Furthermore, Tibetans have been reported to display lower O_2_ consumption for a given exercise workload compared to lowlanders, suggesting a better O_2_ economy (Ge et al., 1994). But how these differences in muscle ultrastructure and O_2_ economy relate to oxidative metabolism in the working muscle has not been investigated. By comparing muscle tissue oxygenation in Tibetans and their Han Chinese counterparts during exercise in normoxia and hypoxia, we aimed to gain further insight into the functional differences in skeletal muscle oxidative metabolism between these two groups.

Near-infrared spectroscopy (NIRS) provides a non-invasive method of assessing changes in muscle tissue oxygenation during exercise (Perrey and Ferrari, 2017). By determining the relative changes in oxy- and deoxy-hemoglobin concentrations ([O_2_Hb] and [HHb], respectively), muscle NIRS signals provide unique insights into O_2_ balance (and therefore oxidative metabolism) in the working muscle (Grassi and Quaresima, 2016; Perrey and Ferrari, 2017). In the brain, NIRS-determined capillary oxygenation is functionally related to the balance between O_2_ saturation of arterial and venous blood, and has been shown to reflect cerebral capillary oxygenation-level-dependent changes (Rasmussen et al., 2007). Studies have reported an association between performance and NIRS-derived cerebral tissue deoxygenation in severe hypoxia (arterial oxygen saturation [SaO_2_] < 75%) during repeated sprints (Smith and Billaut, 2010), incremental exercise (Subudhi et al., 2007; Peltonen et al., 2009), and static maximal or sub-maximal muscle contraction to exhaustion (Rasmussen et al., 2007; Rupp and Perrey, 2009; Vogiatzis et al., 2011; Millet et al., 2012).

It has been speculated that cerebral tissue oxygenation may play a pivotal role in the limitation of performance during exercise in severe hypoxia [(Kjaer et al., 1999; Amann et al., 2006, 2007; Rasmussen et al., 2010), see Fan and Kayser (2016) for review]. During incremental cycling at 3,658 m, Tibetans have been reported to exhibit higher internal carotid blood flow and cerebral O_2_ delivery compared to Han Chinese (Huang et al., 1992). Whether these differences translate to higher cerebral tissue oxygenation, and thereby could partly account for the superior performance in Tibetans is unknown.

The purpose of this study was to compare the muscle and cerebral tissue oxygenation responses to exercise in a pressure-regulated chamber in normobaric normoxia (air at 1 atm, to simulate sea-level) and hypobaric hypoxia (air at 0.5 atm, equivalent to ∼5,000 m altitude) in Tibetans and Han Chinese living in Xining (2,260 m) of the Qinghai Province, People’s Republic of China. We tested the hypothesis that when compared to Han Chinese, Tibetans would exhibit higher brain and muscle tissue oxygenation during exercise in hypoxia, but not during exercise in normoxia.

## Materials and Methods

### Participants

Forty male participants were initially recruited into this study. Following saline-contrast echocardiography screening to exclude participants with a patent foramen ovale (see below), twenty male participants completed this study, consisting of ten Han Chinese and ten Tibetans (Table 1). The Tibetan group consisted of individuals of Tibetan descent who were born and raised at altitudes of >3,500 m and currently residing in Xining of the Qinghai Province, Peoples Republic of China, while the Han Chinese group were all second-generation permanent residents of Xining of the Qinghai Province, Peoples Republic of China. To minimize the confounding influence of training status and physical activity, we recruited recreationally active individuals.

**TABLE 1:** Participant characteristics of Han Chinese and Tibetans (values concern acute normobaric normoxic condition).

	Han Chinese	Tibetan	*p*-value
***n***	**10**	**10**	
_*Age (years)*_	23.3 ± 1.6	21.2 ± 3.1	0.078
_*Height (cm)*_	173.9 ± 6.4	171.0 ± 6.1	0.531
Body mass (kg)	62.2 ± 7.7	64.4 ± 7.7	0.314
BMI (kg m^–2^)	20.5 ± 1.9	22.0 ± 1.9	0.108
V̇O_2_ peak (ml.min^–1^.kg)	35.9 ± 4.1	38.9 ± 10.2	0.408
[Hb] (g.L^–1^)	16.5 ± 1.7	16.0 ± 3.2	0.724
Hct (%)	55.7 ± 2.1	56.2 ± 3.5	0.658

*BMI, body mass index; V̇O_2_ peak, O_2_ uptake at 1 atm; [Hb], Hemoglobin concentration; Hct, hematocrit concentration.*

All the participants were judged healthy by medical history, physical examination, resting electrocardiogram, echocardiogram and respiratory function tests and were not taking any medication. The participants were informed of the experimental procedures and potential risks involved in the study before their written consent was obtained. The study was approved by the University of Oregon Institutional Review Board and the Qinghai High Altitude Medical Science Institutional Committee on Human Research and complied with the *Declaration of Helsinki*.

#### Saline-Contrast Echocardiography Screening

All the participants underwent saline-contrast echocardiography with and without performing a Valsalva maneuver to screen for the presence of patent foramen ovale (PFO). The foramen ovale is an interatrial communication which allows blood flow to bypass the pulmonary circulation during fetal life but normally closes after birth. Importantly, the presence of a PFO in adults has been shown to elevate resting alveolar-arterial O_2_ gradient (i.e., reduced pulmonary gas exchange efficiency) and lower SaO_2_ during maximal exercise in normoxia (Lovering et al., 2011). Likewise, the presence of a PFO is believed to exacerbate arterial hypoxemia in severe chronic obstructive pulmonary disease patients at rest (Allemann et al., 2006; Brenner et al., 2015; Elliott et al., 2015). Therefore, to avoid the possible confounding influence of a PFO, those who were determined to have a PFO were excluded from the study. Similarly as previously found in Sherpas living at high altitude (Foster et al., 2014), the prevalence of PFO was 50% in our cohort of Tibetans.

### Experimental Design

The participants visited the laboratory on three occasions. After a full familiarization with the experimental procedures outlined below (visit one), the participants underwent two experimental exercise sessions (visits two and three) in a pressure-regulated chamber (3 m × 8 m × 3 m hyper- and hypobaric pressure chamber, Shanghai Far East Petroleum Machinery Co., China): (i) normobaric normoxia (simulated sea-level by increasing the chamber pressure to 1 atm), and (ii) hypobaric hypoxia (simulated 5,000 m by decreasing the chamber pressure to 0.5 atm), in a randomized, single-blinded, balanced fashion. Due to technical limitations with the chamber, there was a mild increase in fraction of inspired O_2_ (FIO_2_) from 0.209 to 0.22–0.24 resulting in a mild increase in inspired O_2_ (see methodological considerations).

For the normobaric normoxia and hypobaric hypoxia sessions, the experimental protocol was comprised of: (i) 20 min instrumentation; (ii) 4 min hyperoxic exposure, breathing 100% O_2_ through a face mask, followed by a 3-min washout period to ensure all the variables returned to pre-O_2_ breathing values; (iii) 4 min resting baseline; (iv) step-incremental cycling until exhaustion; (v) 5 min recovery; and (vi) ramp-incremental cycling until exhaustion. All of the experimental sessions were conducted at the National Key Laboratory of High Altitude Medicine in the city of Xining of the Qinghai Province, People’s Republic of China. Before each experimental session, all participants were asked to abstain from caffeine for 12 h, and alcohol for 24 h.

### Exercise Tests

#### Step-Incremental Cycling Until Exhaustion

Seated on a reclining ergometer tilted into a left lateral position (Ergoselect 1000, Ergoline GmbH, Bitz, Germany), the participants were instructed to begin cycling at 70 watts, at a pedaling rate of 70 rpm. The work rate was increased by 30 watts every 3 min thereafter until the participant reached voluntary exhaustion.

#### Ramp Incremental Cycling to Exhaustion

Following a 5-min recovery period whilst positioned on the ergometer, the participants were instructed to begin cycling at 0 watts, at a pedaling rate of 70 rpm. The work rate was increased by 0.5 watts every second (i.e., equivalent of 30 watts.min^–1^) thereafter until the participant reached voluntary exhaustion.

### Measurements

#### Respiratory Variables

Throughout the experimental protocol, the participants wore a facemask attached to a spirometer (TripleV-volume sensor, CareFusion, San Diego, CA, United States), from which expired gases and breath-by-breath respiratory flow was monitored using a metabolic cart (Jaeger, CareFusion, San Diego, CA, United States). Ventilation (V̇E), O_2_ uptake (V̇O_2_), expired CO_2_ (V̇CO_2_), and respiratory exchange ratio (RER) were then calculated by the metabolic cart and expressed in either L.min^–1^ BTPS (for V̇E) or mL.min^–1^ STPD (V̇O_2_ and V̇CO_2_).

#### Cardiovascular and Cerebrovascular Variables

Continuous beat-to-beat blood pressure was monitored using finger plethysmography (Finometer MIDI, Finapres Medical Systems, Amsterdam, Netherlands), from which mean blood pressure (BP) was derived from the timed-average of the BP waveform. Peripheral O_2_ saturation (SpO_2_) was measured from the right side of the forehead using pulse oximetry (N-200, Nellcor Inc., Hayward, CA, United States). A three-lead electrocardiogram was used to determine heart rate (HR). Stroke volume (SV) was estimated using transthoracic electrical bioimpedance cardiography (PhysioFlow^®^, Manatec PF07 Enduro, Paris, France). Cardiac output (CO) was subsequently calculated by multiplying SV with HR.

Bilateral middle cerebral artery blood velocities (MCAv, an index of cerebral blood flow) were measured in the middle cerebral artery using a 2-MHz pulsed Doppler ultrasound system (ST3, Spencer Technology, Seattle, OR, United States). The Doppler ultrasound probes were positioned over the temporal windows and held in place with an adjustable plastic headband. The MCAv signals were acquired at depths ranging from 43 to 54 mm. Signal quality was optimized, and an M-mode screen shot was recorded to facilitate subsequent probe placements. In our hands, day-to-day reproducibility of MCAv has a coefficient of variation of <10%. The bilateral MCAv were averaged using the following equation to represent global cerebral blood flow (CBF) during rest and exercise:

(1) $$\mathrm{mean}\,\mathrm{MCAv}=\,\left[\frac{\mathrm{left}\,\mathrm{MCAv}+\mathrm{right}\,\mathrm{MCAv}}{2}\right]$$

#### Muscle and Cerebral Tissue Oxygenation

Muscle tissue oxygenation in the left vastus lateralis muscle (∼15 cm proximal and 5 cm lateral to the superior border of the patella) was measured by monitoring changes in [O_2_Hb] and [HHb] concentrations obtained using spatially resolved, continuous wave near-infrared spectroscopy (NIRS, Oxymon MKIII, Artinis, Zetten, Netherlands). For the muscle tissue oxygenation, a source-detector spacing of 3.8 cm and a differential pathlength factor of 4.0 were used (Duncan et al., 1995). Cerebral tissue oxygenation in the left prefrontal lobe was assessed with an additional NIRS channel of the same instrument. Both muscle and cerebral NIRS channels were zeroed at rest and expressed as absolute values. For the cerebral tissue oxygenation, source-detector spacing was set at 4.1 cm and data obtained from the optodes were used to calculate changes in [O_2_Hb] and [HHb] with a differential pathlength factor (DPF) calculated using the formula: DPF = 4.99 + 0.067 × age^0.814^ (Duncan et al., 1995). Total Hb ([totHb]) was calculated using the equation:

(2) [totHb]=[O⁢Hb2]+[HHb]

The cerebral and muscle [O_2_Hb], [HHb], and [totHb] are expressed as absolute values (μMol).

#### Arterial Blood Gases

Arterial blood gas samples were obtained from a 22-gauge arterial catheter placed into a radial artery; blood samples (2 mL) were taken over approximately five cardiac cycle periods into a pre-heparinized syringe. Following standardized calibration, all blood samples were analyzed using an arterial blood-gas analyzing system (ABL 77 Sci, Radiometer, Copenhagen, Denmark) for pH, partial pressure of arterial O_2_ (PaO_2_) and CO_2_ (PaCO_2_), arterial O_2_ saturation (SaO_2_), hemoglobin concentration ([Hb]) and hematocrit (Hct). Arterial O_2_ content (CaO_2_) was subsequently calculated using the equation:

(3) CaO=2[Hb]×1.36×[SaO2100]+PaO×20.003

Aural temperature from the right ear was recorded as a surrogate of core body temperature. The blood gas values were temperature corrected (Kelman and Nunn, 1966; Severinghaus, 1966).

### Energy Economy

Energy expended (EE) was calculated using the equation (Moseley and Jeukendrup, 2001):

$$\text{EE}=\left[\left(\dot{\text{V}}\,{\text{O}}_{2}\times 3.869\right)+\left(\dot{\text{V}}\,{\mathrm{CO}}_{2}\times 1.195\right)\times \left(4.186/60\right)\times 1000\right]$$

where, EE is energy expended in J.s^–1^, V̇O_2_ is O_2_ uptake in L.min^–1^ and V̇CO_2_ is the CO_2_ expired in L.min^–1^.

Gross efficiency (GE) during steady-state exercise was calculated using the equation (Moseley and Jeukendrup, 2001):

(4) GE=Work⁢rate/EE×100

Where GE is gross efficiency in percentage, work rate is in watts, and EE is energy expended in J.s^–1^.

Exercise economy (EC) was calculated as power output divided by O_2_ uptake and expressed as kJ.L^–1^.

Except for V̇E, V̇O_2_, V̇CO_2_ and RER which were recorded on the metabolic cart, all analog data were sampled and recorded at 200 Hz on a computer for off-line analysis (Powerlab 16/30, ADInstruments, Dunedin, New Zealand).

### Statistical Analysis

Unpaired *t* test with Welch’s correction was used to compare the participants’ characteristics between Han Chinese and Tibetan (Prism 8, GraphPad Software, San Diego, CA, United States). Analyses of resting parameters were performed on averaged data from the last 2 min of the baseline period, and from the last 1 min of the hyperoxic exposure and recovery period. During exercise, the mean values during the last 30 s of workload (i.e., 70 watts, 100 watts, 130 watts, etc.), and the mean value of the last 30 s of the ramp incremental exercise for maximal exercise effort (MAX) were extracted for analysis.

The main effects of experimental condition (normoxia vs. hypoxia) and group (Han Chinese vs. Tibetans) on arterial blood gases, cardiorespiratory variables, cerebral haemodynamics and muscle tissue oxygenation during 100% O_2_ breathing, at rest, during steady-state exercise (70 watts, 100 watts, 130 watts, and 160 watts), recovery and maximal exercise effort were assessed using mixed linear model analysis (SPSS Statistics version 23, IBM Corporation, Armonk, NY, United States). For significant effects and interaction between hypoxia effect and group effect, *post hoc* tests were performed using Sidak’s adjustment for multiple comparisons (*α*-level of 0.05). In addition to *p*-values, Cohen’s *d* values (effect size) are reported for altitude and group effects. Cohen’s *d* value was calculated using the formula (Cohen, 1977):

(5) $$d=\left[\frac{{\text{M}}_{1\,}-{\text{M}}_{2}}{\sigma_{\mathrm{pooled}}}\right]$$

where, M_1_ and M_2_ are means of group 1 and 2; σ_*pooled*_ is the standard deviation of the pooled data. The effect sizes were classified as (Sullivan and Feinn, 2012): negligible (*d* < 0.2); small (*d* ≥ 0.2); medium (*d* ≥ 0.5); large (*d* ≥ 0.8); and very large (*d* ≥ 1.3). Data are reported as mean ± SD in text, tables and figures.

## Results

### Hyperoxia

In hypoxic conditions, 100% O_2_ breathing increased muscle [O_2_Hb] by ∼2.9 μM in both groups (*F* = 7.8, *d* = 0.8, *p* = 0.012), while neither muscle [HHb] (*F* = 1.3, *p* = 0.264) nor [totHb] were affected (*F* = 1.2, *p* = 0.275). There was a tendency for muscle [HHb] to be lower by ∼2.1 μM in Tibetans during 100% O_2_ breathing (*F* = 3.5, *d* = 0.4, *p* = 0.071 vs. Han Chinese, Figure 4). In both groups, 100% O_2_ breathing decreased MCAv by ∼7.3 cm.s^–1^ in hypoxia compared to normoxia (*F* = 5.4, *d* = 0.5, *p* = 0.032), while no significant group effects were observed (*p* > 0.05).

#### Performance

**FIGURE 1:**
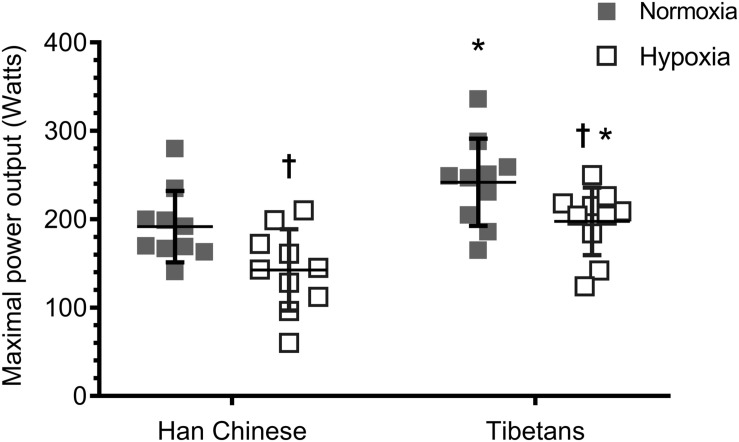
Aerobic capacity during ramp incremental cycling to exhaustion in Han Chinese and Tibetans. *different from Han Chinese, *p* < 0.05; ^†^different from normoxia. Data expressed mean ± SD.

Hypoxia reduced aerobic capacity by ∼22% in both Han Chinese and Tibetans compared to their normoxic values (*F* = 31.5, *d* = 0.8, *p* < 0.001, Figure 1). Irrespective of the experimental condition, Tibetans performed significantly better than their Han Chinese counterparts by ∼32% during incremental exercise to exhaustion (*F* = 8.8, *d* = 1.0, *p* = 0.008).

### Resting, Steady-State Exercise and Recovery

#### Arterial Blood Gases

At rest, lowering barometric pressure reduced PaO_2_ by ∼85.6 mmHg (*F* = 1778.1), PaCO_2_ by ∼6.3 mmHg (*F* = 112.7), SaO_2_ by ∼16.8% (*F* = 132.8) and CaO_2_ by ∼3.0 mL O_2_.dl^–1^ (*F* = 10.51), and elevated resting pH by ∼0.05 (*F* = 60.5) in both Han Chinese and Tibetans (*d* > 1.0 and *p* < 0.01 vs. normoxia for all, Table 2).

**TABLE 2:** Resting cardiorespiratory, cerebral haemodynamics and muscle tissue oxygenation in Han Chinese and Tibetans in normobaric normoxia and hypobaric hypoxia.

	Han Chinese		Tibetan		Main effects (*p*-values)		
*n*	10		10				
		
*Arterial blood gases*	Normoxia	Hypoxia	Normoxia	Hypoxia	Condition	Group	Interaction
PaO_2_ (mmHg)	128.8 ± 7.5	45.6 ± 4.9†	135.1 ± 8.9	47.2 ± 7.3†	**0.000**	0.158	0.263
PaCO_2_ (mmHg)	40.2 ± 5.2	33.0 ± 3.8†	37.7 ± 4.2	32.4 ± 4.1†	**0.000**	0.417	0.126
pH	7.35 ± 0.02	7.41 ± 0.03†	7.37 ± 0.03	7.41 ± 0.03†	**0.000**	0.319	0.091
SaO_2_ (%)	98.8 ± 0.2	81.2 ± 4.0†	99.0 ± 0.1	83.0 ± 8.4†	**0.001**	0.503	0.613
CaO_2_ (mL O_2_.dl^–1^)	22.2 ± 2.3	19.3 ± 2.0†	21.7 ± 4.2	18.6 ± 2.6†	**0.005**	0.518	0.879
***Cardiorespiratory***							
BP (mmHg)	105.7 ± 8.8	102.3 ± 8.2	103.8 ± 10.3	99.3 ± 8.8	0.177	0.396	0.856
HR (b.min^–1^)	74 ± 11	90 ± 13†	72 ± 12	95 ± 19†	**0.001**	0.729	0.229
CO (L.min^–1^)	7.3 ± 1.3	7.1 ± 1.3†	8.1 ± 1.9	8.3 ± 1.7†	**0.024**	0.959	0.708
V̇E (L.min^–1^)	17.3 ± 2.9	14.7 ± 1.5†	17.9 ± 2.6	16.1 ± 2.2†	**0.005**	0.276	0.550
V̇O_2_ (ml.min^–1^)	365 ± 40	376 ± 56	347 ± 45	383 ± 57	0.167	0.658	0.328
V̇CO_2_ (ml.min^–1^)	340 ± 46	323 ± 43	304 ± 41	337 ± 53	0.579	0.481	0.099
RER	0.92 ± 0.05	0.86 ± 0.07†	0.88 ± 0.04	0.88 ± 0.05	0.060	0.517	**0.047**
SpO_2_ (%)	98.8 ± 1.1	78.4 ± 8.0†	99.0 ± 0.7	83.9 ± 4.3†	**0.001**	0.054	0.074
***Cerebral haemodynamics***							
MCAv (cm.s^–1^)	63.1 ± 16.9	65.2 ± 13.6	54.4 ± 9.6	58.3 ± 8.6	0.302	0.134	0.808
Cerebral [O_2_Hb] (μmol)	0.9 ± 3.0	0.3 ± 2.4	1.4 ± 2.6	1.7 ± 3.8	0.321	0.888	0.649
Cerebral [HHb] (μmol)	−0.2 ± 1.5	−0.5 ± 2.2†	2.4 ± 3.9	2.2 ± 3.0†	**0.005**	0.793	0.956
Cerebral [totHb] (μmol)	0.7 ± 3.7	−0.1 ± 4.1†	3.8 ± 4.7	3.9 ± 5.5†	**0.017**	0.800	0.728
***Muscle oxygenation***							
Muscle [O_2_Hb] (μmol)	1.2 ± 2.3	3.0 ± 2.7	3.4 ± 4.5	1.9 ± 4.7	0.651	0.906	0.174
Muscle [HHb] (μmol)	−0.6 ± 3.6	−0.5 ± 2.5†	1.9 ± 3.3	1.8 ± 2.5†	**0.016**	0.992	0.876
Muscle [totHb] (μmol)	0.6 ± 3.4	2.5 ± 3.2	5.3 ± 7.2	3.7 ± 5.1	0.070	0.924	0.265

*PaO_2_, partial pressure of arterial O_2_; PaCO_2_, partial pressure of arterial CO_2_; SaO_2_, arterial O_2_ saturation; CaO_2_, arterial O_2_ content; BP, blood pressure; HR, heart rate; CO, cardiac output; V̇E, pulmonary ventilation; V̇O_2_, O_2_ uptake; V̇CO_2_, expired CO_2_; RER, respiratory exchange ratio; SpO_2_, peripheral O_2_ saturation; MCAv, middle cerebral artery velocity; [O_2_Hb], oxy-hemoglobin concentration; [HHb], deoxy-hemoglobin concentration; [totHb], total hemoglobin concentration. Values are mean ± SD. ^†^different from normoxia (*p* < 0.05). Bold text indicate significant main effect (*p* < 0.05).*

On average, hypoxia reduced PaO_2_ by ∼76.3 mmHg throughout step-incremental cycling (*F* = 8682.7, *d* = 2.0), PaCO_2_ by ∼13.7 mmHg (*F* = 554.4, *d* = 1.5), SaO_2_ by ∼17.5% (*F* = 2240.9, *d* = 1.0), CaO_2_ by ∼3.2 mL O_2_.dl^–1^ (*F* = 81.8, *d* = 1.1) in both Han Chinese and Tibetans, while it elevated [Hb] by ∼0.8 g.L^–1^ (*F* = 9.1, *d* = 0.5) and pH by ∼0.09 (*F* = 92.9, *d* = 1.5, *p* < 0.001 for all, Table 3). There was no other difference between Han Chinese and Tibetans in any of the arterial blood gas parameters during exercise in both hypoxic and normoxic conditions (*p* > 0.05, Table 3).

**TABLE 3:** Arterial blood gases in Han Chinese and Tibetans during exercise at simulated sea-level and 5,000 m.

		Han Chinese		Tibetan		Main effects (*p*-values)		
*n*		10		10				
				
		Normoxia	Hypoxia	Normoxia	Hypoxia	Condition	Group	Interaction
PaO_2_ (mmHg)	70 W	128.8 ± 7.5	45.4 ± 2.9†	126.9 ± 10.1	46.3 ± 4.6†	**0.001**	0.474	0.131
	100 W	121.9 ± 9.3	47.3 ± 5.2†	124.9 ± 7.6	46.9 ± 3.8†			
	130 W	120.8 ± 8.0	−	124.2 ± 10.2	48.0 ± 4.5†			
	160 W	−	−	122.0 ± 8.9	−			
	Max	124.3 ± 8.4	50.8 ± 4.6†	122.9 ± 9.5	49.5 ± 3.5†	**0.001**	0.658	0.962
PaCO_2_ (mmHg)	70 W	46.2 ± 7.1	33.2 ± 4.6†	42.7 ± 4.7	31.8 ± 4.0†	**0.001**	0.575	0.160
	100 W	45.5 ± 7.7	28.5 ± 5.3†	43.4 ± 4.9	29.5 ± 4.4†			
	130 W	47.0 ± 5.6	−	42.2 ± 5.9	25.5 ± 4.1†			
	160 W	−	−	42.5 ± 6.2	−			
	Max	39.1 ± 5.5	22.9 ± 3.3†	41.8 ± 5.8	24.1 ± 4.3†	**0.001**	0.310	0.592
pH (a.u.)	70 W	7.30 ± 0.04	7.36 ± 0.03†	7.32 ± 0.03	7.38 ± 0.04†	**0.001**	0.303	0.623
	100 W	7.26 ± 0.05	7.33 ± 0.04†	7.29 ± 0.03	7.35 ± 0.02†			
	130 W	7.22 ± 0.07	−	7.26 ± 0.03	7.31 ± 0.03†			
	160 W	−	−	7.22 ± 0.04	−			
	Max	7.19 ± 0.06	7.29 ± 0.03†	7.19 ± 0.05	7.28 ± 0.04†	**0.001**	0.988	0.954
SaO_2_ (%)	70 W	98.2 ± 0.4	79.7 ± 2.5†	98.6 ± 0.3	81.9 ± 4.5†	**0.001**	0.286	0.121
	100 W	98.0 ± 0.6	80.0 ± 4.6†	98.4 ± 0.2	81.4 ± 2.8†			
	130 W	97.6 ± 0.7	−	98.2 ± 0.3	81.1 ± 3.7†			
	160 W	−	−	97.9 ± 0.3	−			
	Max	97.6 ± 0.8	81.2 ± 4.7†	97.6 ± 0.9	80.5 ± 3.2†	**0.001**	0.804	0.752
[Hb] (g.L^–1^)	70 W	16.3 ± 2.5	17.6 ± 1.0	16.2 ± 2.5	16.6 ± 1.2	**0.003**	0.257	0.190
	100 W	17.0 ± 2.1	18.3 ± 2.0	16.1 ± 2.3	16.8 ± 1.2			
	130 W	17.1 ± 1.8	−	16.9 ± 1.1	17.7 ± 0.8			
	160 W	−	−	17.3 ± 1.4	−			
	Max	17.8 ± 2.5	19.3 ± 1.7	17.2 ± 1.8	18.0 ± 1.1	0.057	0.189	0.543
CaO_2_ (ml O_2_.dl^–1^)	70 W	21.9 ± 3.3	19.0 ± 1.4†	21.8 ± 3.2	18.3 ± 2.0†	**0.001**	0.443	0.488
	100 W	22.7 ± 2.7	19.9 ± 3.1†	21.6 ± 3.0	18.5 ± 1.5†			
	130 W	22.8 ± 2.3	−	22.6 ± 1.4	19.3 ± 1.2†			
	160 W	−	−	23.0 ± 1.8	−			
	Max	23.6 ± 3.2	21.2 ± 2.5†	22.9 ± 2.2	19.6 ± 1.2†	**0.001**	0.163	0.623

*PaO_2_, partial pressure of arterial O_2_; PaCO_2_, partial pressure of arterial CO_2_; SaO_2_, arterial O_2_ saturation; [Hb], hemoglobin concentration; CaO_2_, arterial O_2_ content. Values are mean ± SD. ^†^different from normoxia (*p* < 0.05). Bold text indicate significant main effect (*p* < 0.05).*

During maximal effort, hypoxia lowered PaO_2_ by ∼72.8 mmHg (*F* = 1569.0, *d* = 1.9), PaCO_2_ by ∼16.7 mmHg (*F* = 259.2, *d* = 1.7), SaO_2_ by ∼16.6% (*F* = 66.1, *d* = 1.9), CaO_2_ by ∼2.9 mL O_2_.dl^–1^ in both groups (*F* = 12.3, *d* = 1.0), while pH was elevated by ∼0.10 compared to normoxia (*F* = 66.1, *d* = 1.7, *p* < 0.001 for all, Table 3). There were no group effects on arterial blood gas parameters (*p* > 0.05, Table 3).

#### Cardiorespiratory

In both Han Chinese and Tibetans, exposure to hypoxia elevated resting HR by ∼19.6 b.min^–1^ (*F* = 58.5, *d* = 1.2), CO by ∼1.0 L.min^–1^ (*F* = 6.1, *d* = 0.6) and lowered SpO_2_ by ∼17.7% (*F* = 152.8, *d* = 1.8) compared to normoxia (*p* < 0.05 vs. normoxia for all, Table 2). There was a trend for Tibetans to display higher resting SpO_2_ compared to the Han Chinese group (*F* = 3.9, *p* = 0.054), particularly in hypoxia (interaction: *F* = 3.4, *p* = 0.074, Table 2). *Post hoc* pairwise analysis showed resting SpO_2_ was higher in Tibetans by ∼5.5% in hypoxia compared to Han (*d* = 0.8, *p* = 0.011), but was not different in normoxia (*p* = 0.917). Hypoxia selectively lowered resting RER in Han Chinese (interaction: *F* = 4.6, *p* = 0.047). As a result, hypoxia lowered resting RER in Han Chinese by ∼0.054 (*d* = 0.8, *p* = 0.009 vs. normoxia), while no change was observed in Tibetans (*d* < 0.1, *p* = 0.929).

**FIGURE 2:**
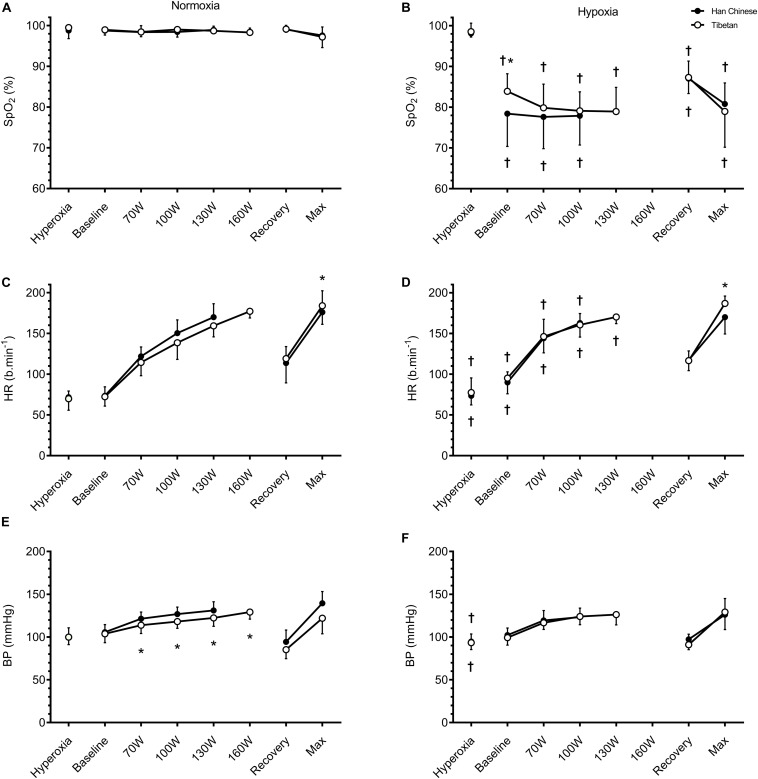
Cardiovascular responses during hyperoxia, baseline, step-incremental cycling, recovery and ramp-incremental cycling to exhaustion (Max) in Han Chinese and Tibetans. Left panel, normoxia: Right panel, hypoxia (equivalent of ∼5,000 m altitude). SpO_2_, peripheral O_2_ saturation; HR, heart rate; BP, blood pressure. *different from Han Chinese, *p* < 0.05; ^†^different from normoxia, *p* < 0.05. Data expressed mean ± SD.

**FIGURE 3:**
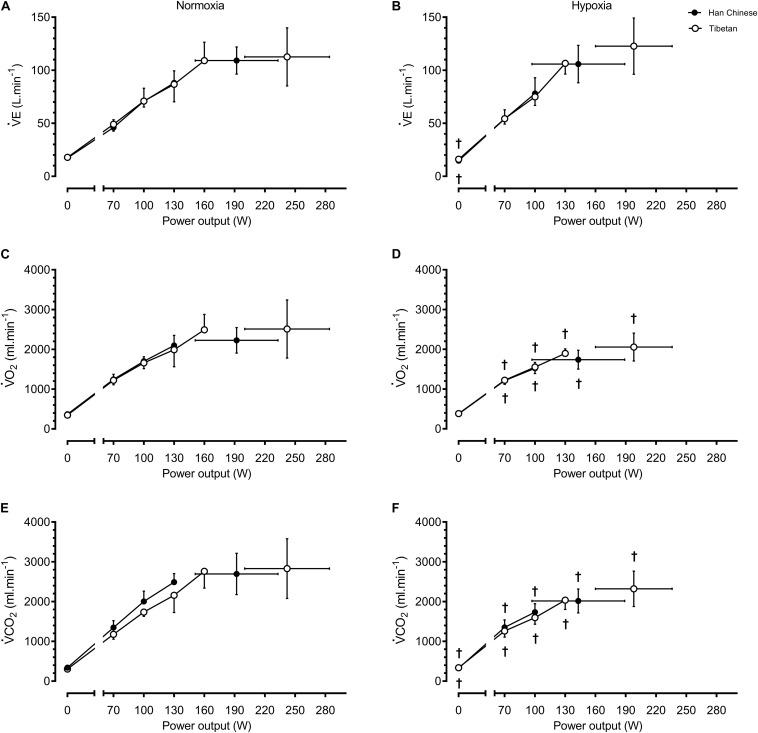
Ventilation and metabolic responses during baseline, step-incremental cycling and maximal exertion (MAX) in Han Chinese and Tibetans. Left panel, normoxia: Right panel, hypoxia (equivalent of ∼5,000 m altitude). V̇E, pulmonary ventilation; V̇O_2_, O_2_ uptake; V̇CO_2_, expired CO_2_. *different from Han Chinese, *p* < 0.05; ^†^different from normoxia l, *p* < 0.05. Data expressed mean ± SD.

For a given workload, hypoxia elevated HR by ∼10.2 b.min^–1^ (*F* = 5.8, *d* = 0.5, *p* = 0.017) and lowered SpO_2_ by ∼20.1% (*F* = 965.9, *d* = 1.5, *p* < 0.001), V̇O_2_ by ∼283 ml.min^–1^ (*F* = 11.9, *d* = 0.6, *p* = 0.001) and V̇CO_2_ by ∼251 ml.min^–1^ (*F* = 80.1, *d* = 0.5, *p* < 0.001), but had no effects on CO or BP (*F* ≤ 0.5, *p* > 0.05, Figures 2, 3). We observed no significant group effects on HR, SpO_2_, V̇O_2_ or CO during step-incremental cycling (*p* > 0.05). There was a group difference on RER during step-incremental cycling (*F* = 9.4, *p* = 0.002), which was mediated by a non-significant group difference in V̇CO_2_ (*F* = 6.0, *d* = 0.2, *p* = 0.054). In both normoxia and hypoxia, RER was lower in Tibetans by ∼0.097 (*d* = 1.1) throughout the step-incremental cycling compared to Han Chinese.

**FIGURE 4:**
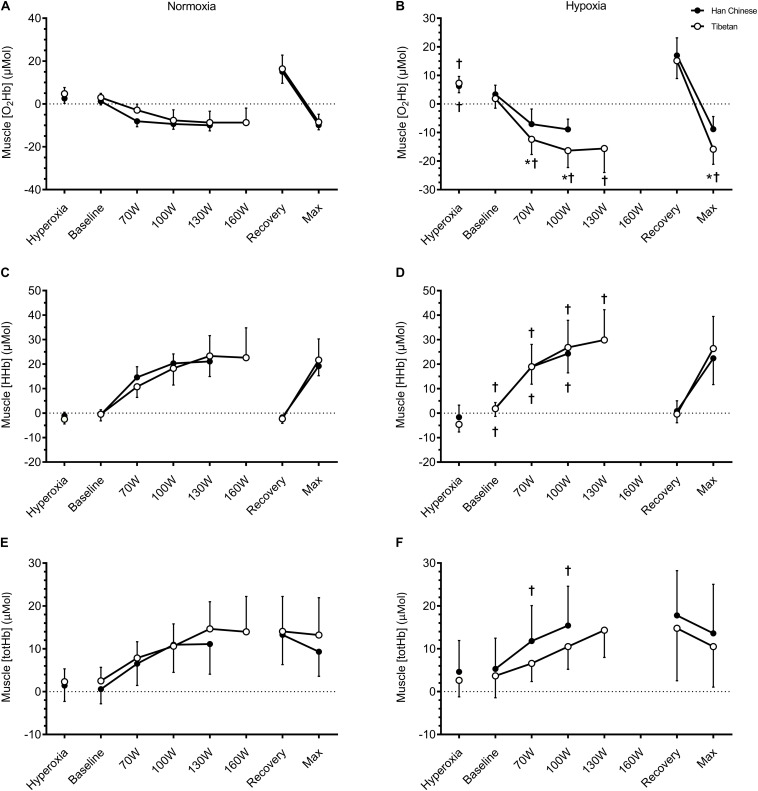
Muscle tissue oxygenation of the vastus lateralis during hyperoxia, baseline, step-incremental cycling, recovery and ramp-incremental cycling to exhaustion (Max) in Han Chinese and Tibetans. Left panel, normoxia: Right panel, hypoxia (equivalent of ∼5,000 m altitude). *different from Han Chinese, *p* < 0.05; ^†^different from normoxia, *p* < 0.05. Data expressed mean ± SD.

During recovery, hypoxia lowered SpO_2_ by ∼11.9% (*F* = 161.2, *d* = 1.8, *p* < 0.001) compared to normoxia, but had no effects on HR (*F* = 0.0, *p* = 0.872) or BP (*F* = 1.3, *p* = 0.269, Figure 2). There were no group differences in cardiorespiratory parameters during recovery (*p* > 0.05).

During maximal effort, hypoxia lowered SpO_2_ by ∼15.6% (*F* = 62.5, *d* = 1.5, *p* < 0.001), V̇O_2_ by ∼462 ml.min^–1^ (*F* = 18.2, *d* = 0.9, *p* = 0.001)), V̇CO_2_ by ∼462 ml.min^–1^ (*F* = 15.9, *d* = 0.8, *p* < 0.001) and CO by ∼1.7 L.min^–1^ (*F* = 5.5, *d* = 0.6, *p* = 0.031), but had no effect on HR or BP (*p* > 0.05, Figure 2, 3). Irrespective of the experimental conditions, Tibetans had higher HR (by ∼17.5 b.min^–1^, *F* = 6.3, *p* = 0.017, Figure 2) and CO at maximal exercise compared to Han Chinese (by ∼3.2 L.min^–1^, *F* = 13.6, *d* = 1.1, *p* = 0.002).

#### Energy Economy

No condition or group effects were observed in EE, GE or EC during step-incremental cycling (*p* < 0.05, Table 4). Hypoxia lowered EE at maximal effort by ∼370 J.s^–1^ (*F* = 10.5, *d* = 1.9, *p* = 0.005). Irrespective of the condition, GE was higher by ∼5%, in Tibetans at maximal effort compared to Han Chinese (*F* = 10.7, *d* = 1.1, *p* = 0.004), while EC was higher by ∼0.18 kJ.L^–1^ (*F* = 5.3, *d* = 0.9, *p* = 0.034, Table 4).

**TABLE 4:** Energy economy in Han Chinese and Tibetans during exercise at simulated sea-level and 5,000 m.

		Han Chinese		Tibetan		Main effects (*p*-values)		
*n*		10		10				
				
		Normoxia	Hypoxia	Normoxia	Hypoxia	Condition	Group	Interaction
EE (J.s^–1^)	70 W	448 ± 46	440 ± 40	428 ± 40	435 ± 24	0.245	0.221	0.337
	100 W	626 ± 43	555 ± 50	592 ± 46	552 ± 42			
	130 W	773 ± 82	–	717 ± 149	681 ± 48			
	160 W	–	–	903 ± 136	–			
	Max	829 ± 133	643 ± 81 †	924 ± 240	751 ± 129	**0.005**	0.958	0.238
GE (%)	70 W	14.9 ± 1.7	14.9 ± 1.8	16.5 ± 1.6	16.1 ± 1.4	0.129	0.397	0.494
	100 W	14.3 ± 1.1	16.6 ± 2.1	17.0 ± 1.4	18.2 ± 1.4			
	130 W	14.9 ± 1.8	–	19.1 ± 5.2	19.2 ± 1.2			
	160 W	–	–	18.2 ± 3.7	–			
	Max	23.1 ± 2.7	24.1 ± 4.2	26.7 ± 3.8*	26.6 ± 4.1*	0.120	**0.004**	0.823
EC (kJ.L^–1^)	70 W	0.81 ± 0.09	0.83 ± 0.06	0.83 ± 0.08	0.82 ± 0.04	0.875	0.398	0.671
	100 W	0.85 ± 0.06	0.95 ± 0.09	0.87 ± 0.08	0.93 ± 0.07			
	130 W	0.90 ± 0.11	–	0.99 ± 0.28	0.99 ± 0.06			
	160 W	–	–	0.96 ± 0.21	–			
	Max	1.22 ± 0.15	1.26 ± 0.22	1.39 ± 0.18*	1.43 ± 0.23*	0.468	**0.034**	0.773

EE, energy expenditure; GE, gross efficiency; EC, exercise economy. Values are mean ± SD. ^†^different from normoxia (*p* < 0.05). *different from Han Chinese (*p* < 0.05). Bold text indicate significant main effect (*p* < 0.05).

#### Muscle Tissue Oxygenation

In both groups, hypobaric hypoxia elevated resting muscle [HHb] by ∼2.4 μM (*F* = 6.4, *d* = 0.8, *p* = 0.016), but had no effects on muscle [totHb] (*F* = 3.4, *p* = 0.070) or [O_2_Hb] (*F* = 0.2, *p* = 0.651). No group effects were observed in resting muscle NIRS signals (*p* > 0.05, Table 2).

During step-incremental cycling, hypoxia lowered muscle [O_2_Hb] by ∼3.1 μM in both groups (*F* = 10.7, *d* = 0.5, *p* < 0.001), and elevated muscle [HHb] by ∼4.7 μM (*F* = 15.1, *d* = 0.5, *p* < 0.001), while muscle [totHb] was unchanged (*F* = 2.6, *p* = 0.108, Figure 4). This hypoxia effect on exercising muscle [O_2_Hb] appeared to be limited to Tibetans (interaction: *F* = 23.3, *p* < 0.001). Hypoxia lowered muscle [O_2_Hb] by ∼7.6 μM in Tibetans (*d* = 1.0, *p* < 0.001 vs. normoxia) but not in Han Chinese (*d* = 0.3, *p* = 0.298, Figure 4). As a result, muscle O_2_Hb was selectively lower in Tibetans during hypoxic exercise (by ∼6.7 μM, *d* = 0.9, *p* = 0.003 vs. Han Chinese), but not during normoxic exercise (*d* = 0.4, *p* = 0.240, Figure 4).

We observed an interaction between hypoxia and group on muscle [totHb] (*F* = 8.7, *p* = 0.004). *Post hoc* analysis showed that hypoxia selectively increased muscle [totHb] in Han Chinese (by ∼4.6 μM, *d* = 0.6, *p* = 0.003), but not in Tibetans (*d* = 0.2, *p* = 0.325, Figure 4). Nevertheless, this increase did not result in a significant difference in muscle [totHb] between the groups during exercise in hypoxia (*d* = 0.5, *p* = 0.117). We found no significant group effects on muscle [HHb] during step-incremental cycling (*F* = 0.1, *p* = 0.778, Figure 4). No significant condition or group effects were observed on muscle tissue NIRS signals during recovery or at maximal effort (*p* > 0.05).

#### Cerebral Tissue Oxygenation and Hemodynamics

When compared to normoxia, hypoxia lowered resting cerebral [HHb] in both groups by ∼2.6 μM (*F* = 10.3, *d* = 0.9, *p* = 0.005) and [totHb] by ∼3.5 μM (*F* = 6.9, *d* = 0.7, *p* = 0.017), but had no effect on resting MCAv (*F* = 1.1, *p* = 0.302) or cerebral [O_2_Hb] (*F* = 1.0, *p* = 0.321, Table 2). No group effects were observed in resting cerebral haemodynamics (*p* > 0.05, Table 2).

**FIGURE 5:**
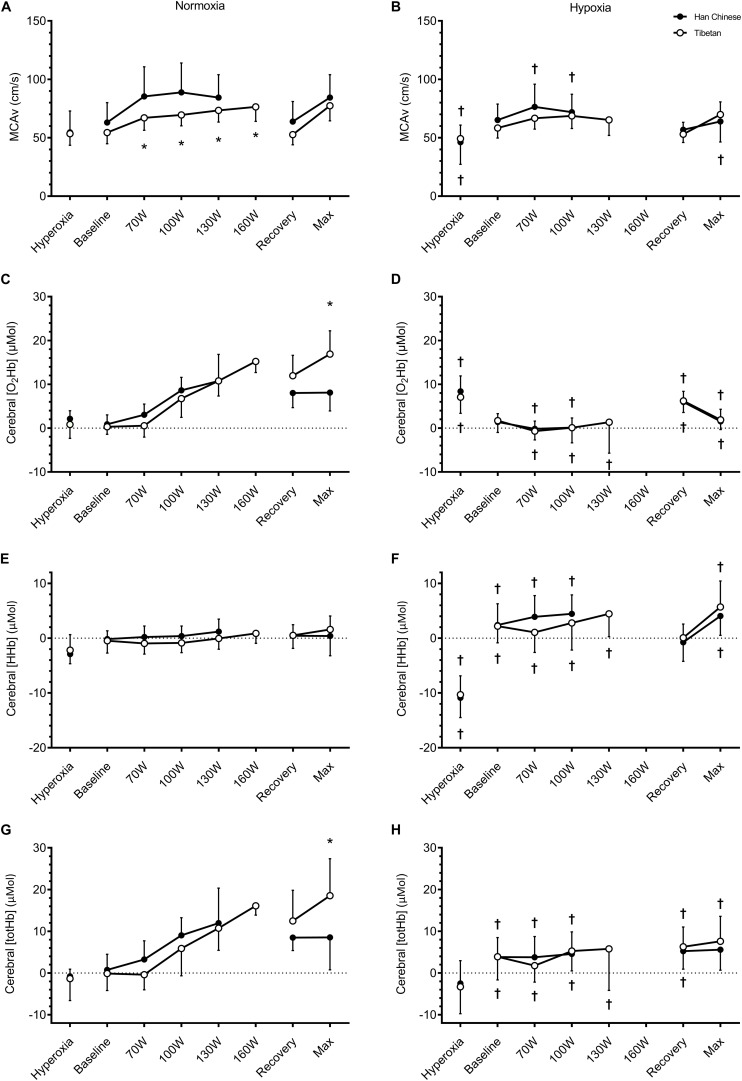
Cerebral tissue oxygenation of the prefrontal cortex during hyperoxia, baseline, step-incremental cycling, recovery and ramp-incremental cycling to exhaustion (Max) in Han Chinese and Tibetans. Left panel, normoxia: Right panel, hypoxia (equivalent of ∼5,000 m altitude). *different from Han Chinese, *p* < 0.05; ^†^different from normoxia, *p* < 0.05. Data expressed mean ± SD.

On average, hypoxia elevated cerebral [HHb] during step-incremental cycling by ∼3.7 μM in both Tibetan and Han Chinese (*F* = 58.4, *d* = 1.8), and lowered cerebral [O_2_Hb] by ∼7.9 μM (*F* = 67.8, *d* = 1.6) and cerebral [totHb] by ∼4.1 μM (*F* = 12.4, *d* = 0.6, *p* < 0.001 for all, Figure 5). Hypoxia also lowered MCAv by ∼8.4 cm.s^–1^ (*F* = 29.6, *d* = 0.5, *p* < 0.001), this reduction was greater in the Han Chinese group (interaction: *F* = 8.0, *p* = 0.006, Figure 5). In Han Chinese, hypoxia lowered MCAv by ∼12.8 cm.s^–1^ during step-incremental cycling (*d* = 0.7, *p* < 0.001), while hypoxia only tended to lower MCAv in Tibetans by ∼4.1 cm.s^–1^ (*d* = 0.4, *p* = 0.055). Tibetans had lower MCAv compared to Han Chinese in normoxia (by ∼15.3 cm.s^–1^, *d* = 0.8, *p* = 0.039), while they were not significantly different in hypoxia (*d* = 0.5, *p* = 0.360, Figure 5). We did not observe any significant group effects on cerebral [O_2_Hb] (*F* = 0.1, *p* = 0.766), [HHb] (*F* = 2.1, *p* = 0.164) or [totHb] during step-incremental cycling (*F* = 0.6, *p* = 0.438, Figure 5).

Hypoxia lowered cerebral [O_2_Hb] during recovery, by ∼3.9 μM in both groups (*F* = 7.3, *d* = 0.8, *p* = 0.011 vs. normoxia) and cerebral [totHb] by ∼4.3 μM (*F* = 8.6, *d* = 0.8, *p* = 0.006), compared to normoxia but not MCAv (*F* = 2.4, *p* = 0.140) or cerebral [HHb] (*F* = 1.2, *p* = 0.283, Figure 5). We found no between-group differences in cerebral haemodynamics during recovery (*p* > 0.05, Figure 5).

At maximal effort, hypoxia lowered MCAv by ∼13.8 cm.s^–1^ (*F* = 17.6, *d* = 1.0, *p* < 0.001), cerebral [O_2_Hb] by ∼10.9 μM (*F* = 54.5, *d* = 1.3) and cerebral [totHb] by ∼6.9 μM (*F* = 13.0, *d* = 0.8), and elevated cerebral [HHb] by ∼4.1 μM (*F* = 17.9, *d* = 1.0, *p* < 0.01 for all vs. normoxia, Figure 5). We observed a group difference in cerebral [O_2_Hb] (*F* = 5.6, *p* = 0.031), but only in normoxia (interaction: *F* = 8.6, *p* = 0.010). Compared to Han Chinese, cerebral [O_2_Hb] was higher in Tibetans at maximal effort in normoxia by ∼8.9 μM (*d* = 1.1, *p* = 0.001), but not in hypoxia (*d* = 0.0, *p* = 0.962, Figure 5). Similarly, there was a significant group effect on cerebral [totHb] (*F* = 5.4, *p* = 0.033). As a result, hypoxia reduced cerebral [totHb] during maximal effort in Tibetans by ∼10.8 μM (*d* = 1.2, *p* = 0.001 vs. normoxia), but not in Han Chinese (*d* = 0.4, *p* = 0.285, Figure 5). No group effect was observed in cerebral [HHb] at maximal exercise (*F* = 1.5, *p* = 0.235).

## Discussion

The underlying mechanisms responsible for Tibetans’ superior work capacity at altitude remains unclear. We compared cerebral and muscle tissue oxygenation responses to step-incremental cycling and at maximal exercise during ramp incremental cycling between Tibetans and Han Chinese in normobaric normoxia and hypobaric hypoxia in a pressure-regulated chamber, simulating altitudes equivalent to sea-level and 5,000 m altitude, respectively. Our main findings are that: (1) Irrespective of the condition, Tibetans consistently outperformed their Han Chinese counterparts and exhibited better economy at maximal exertion; (2) In normoxia, Tibetans displayed lower MCAv during submaximal exercise, yet achieved higher cerebral [O_2_Hb] and [totHb] at maximal effort compared to Han Chinese; (3) In hypoxia, Tibetans displayed lower muscle [O_2_Hb] compared to the Han Chinese, which was not mediated by any differences in SaO_2_ or CaO_2_; and (4) For a given workload, Tibetans exhibited greater muscle desaturation during exercise in hypoxia, but not in normoxia. It follows that during exercise in hypoxia Tibetans as compared to Han Chinese seem to defend their brain oxygenation over muscle oxygenation without any obvious cost to performance.

For a given workload, Subudhi et al. (2008) previously found comparable decreases in muscle [O_2_Hb] between exercise at low altitude and at 4,300 m in lowlanders, while muscle [HHb] was increased at high altitude, suggesting greater O_2_ extraction. Hypoxic training has been shown to enhance muscle [HHb] response to hypoxic exercise, while muscle [O_2_Hb] response was unaffected (Wang et al., 2010). Our Han Chinese confirmed these observations by showing unchanged muscle [O_2_Hb] responses to incremental exercise in hypobaric hypoxia when comparing with identical workloads in normoxia (Figure 4). Intriguingly, hypobaric hypoxia elicited a greater reduction in muscle [O_2_Hb] in Tibetans during exercise, while the increase in [HHb] was unaffected (Figure 4B). Since both SaO_2_ and CaO_2_ were similar in Tibetans and Han Chinese during hypoxic exercise (Table 3), this would suggest similar O_2_ extraction in Tibetans during hypoxic exercise (similar changes in [HHb]), but lower muscle perfusion (less [O_2_Hb] and [totHb]). Our muscle NIRS data allude to lower muscle O_2_ utilization in Tibetans for a given workload in hypoxia compared to Han Chinese. Furthermore, Tibetans achieved higher maximal workload despite similar V̇O_2_max in normoxia, and thus exhibited better exercise economy (Table 4). Our data support a previous study by Ge et al. (1994), who reported lower V̇O_2_ consumption in Tibetans for a given submaximal exercise load compared to well-acclimatized (∼3.2 years) Han Chinese at 4,700 m, along with greater maximal work at a lower V̇O_2_max. These findings allude to an enhanced muscle O_2_ economy, presumably due to a metabolic adaptation in Tibetans. Intriguingly, this enhanced muscle O_2_ economy appears to persist following descent to lower altitude (Marconi et al., 2005).

The underlying mechanism for this lower muscle perfusion and O_2_ utilization in Tibetans is unclear. One possible explanation is a higher reliance on glucose oxidation in Tibetans during hypoxic exercise. Glucose oxidation generates higher number of high-Englert phosphate bonds per mole of O_2_ (6.3 unit.mol O_2_^–1^) compared to fatty acid oxidation (4.1 unit.mol O_2_^–1^) (Kessler and Friedman, 1998). Therefore a higher reliance of glucose oxidation coupled with a lower reliance on fat oxidation increases the amount of ATP produced per molecule of O_2_ consumed, thereby enhances work-to-O_2_ ratio in the muscle (Hochachka et al., 1992; Hoppeler and Vogt, 2001). There is a predilection in Tibetans and Sherpas (a Nepalese ethnic group of Tibetan origin) toward carbohydrate oxidation and reduced reliance on intra-myocellular lipids and lipid substrates (Hochachka et al., 1992; Kayser et al., 1996; Ge et al., 2015; Horscroft et al., 2017). The capacity of fatty acid oxidation is typically reduced at high altitude, mediated by decreased 3-hydroxyacyl-CoA dehydrogenase activity (Levett et al., 2012; Horscroft and Murray, 2014). Additional evidence for metabolic adaptation emerged from a genomic scan in Tibetan highlanders, where a haplotype of peroxisome proliferator-activated receptor α (PPARα) which was positively selected and associated with lower hematocrit (Simonson et al., 2010). PPARα encodes the nuclear peroxisome proliferator activated receptor α, which regulates fatty acid metabolism (Cullingford et al., 2002). At rest, we found acute hypoxic exposure selectively lowered RER in Han Chinese, presumably due to a shift toward fat utilization, while it was unchanged in Tibetans (Table 2). As reviewed by Gilbert-Kawai et al. (2014), our findings support a preferential use of carbohydrate-metabolism in Tibetans.

A potential explanation for the ability to extract oxygen to a similar extent despite reduced perfusion and saturation is a reduced muscle diffusion path for O_2_ in Tibetans. In altitude-born Sherpas, Kayser et al. (1991) found muscle fiber cross-sectional area to be smaller, while capillary density was higher when compared to untrained lowlanders, but not different from that of fully acclimatized Caucasian climbers having spent two months at extreme altitude. This combination of smaller fiber cross-sectional area coupled with higher capillary density reduces the diffusion path for O_2_. Since these muscle characteristics were similar between Sherpas and acclimatized Caucasian climbers, it is likely the result of adaptation to extreme altitude *per se*, rather than a unique feature of the Himalayan natives. In addition, lower mitochondrial densities have been observed in both high altitude and lowland-dwelling Sherpas and Tibetans, resulting in a higher maximal O_2_ consumption-to-mitochondrial volume ratio (Kayser et al., 1991, 1996). Collectively, a shortened O_2_ diffusion path and higher mitochondrial O_2_ consumption would facilitate the higher muscle oxidative metabolism in Tibetans during hypoxic exercise.

Hyperventilation-induced hypocapnia during high-intensity exercise causes cerebral vasoconstriction, which can compromise cerebral O_2_ delivery and decrease cerebral tissue oxygenation (Fan and Kayser, 2016). In Han Chinese, we observed a steady rise in MCAv during step-incremental cycling in normoxia, which began to decline during workloads above 100 W (Figure 5A), likely the result from hypocapnia (Table 3). In contrast, Tibetans did not display the expected decline in MCAv at higher exercise intensity in normoxia, despite similar PaCO_2_ values (Table 3). Instead, MCAv, cerebral [O_2_Hb] and [totHb] continued to rise in Tibetans during high intensity normoxic exercise (Figure 5). This difference in MCAv response resulted in higher cerebral [O_2_Hb] and [totHb] in Tibetans at maximal exercise compared to Han Chinese. This finding supports a blunted MCAv response to hypocapnia in Tibetans during heavy intensity exercise. Together with their higher cardiac output, this enabled them to maintain higher prefrontal tissue oxygenation at maximal effort. Since cerebral tissue deoxygenation has been proposed as one of the limiting factors of exercise performance (Nybo and Rasmussen, 2007; Amann and Kayser, 2009), the ability to maintain higher cerebral tissue oxygenation in normoxia could partly account for the superior aerobic performance in Tibetans.

The effect of hypoxia on the cerebral perfusion response to exercise varies between populations. In Tibetans living at 3,658 m, Huang et al. (1992) found a greater increase in internal carotid artery (ICA) blood velocity and estimated cerebral O_2_ delivery during incremental exercise compared to Han Chinese. They found ICA blood velocity returned toward resting values at maximal exercise in the Han Chinese group, while ICA blood velocity remained elevated in Tibetans. During incremental exercise at simulated 5,000 m, we found no difference in MCAv and cerebral NIRS responses between Tibetan and Han Chinese (Figure 5). Compared to their normoxic values, hypoxia lowered MCAv during exercise by ∼23% in Han Chinese, while it only tended to lower it by ∼6% in Tibetans. As a result, the between-group difference in MCAv and cerebral NIRS responses to normoxic exercise were abolished during exercise in hypoxia. Together with the findings by Huang et al. (1992), our data indicates that Tibetans have a blunted cerebrovascular response to hypoxia compared to their Han Chinese counterparts, without adversely lowering cerebral tissue oxygenation.

We previously found higher SpO_2_ (∼9%) and HR (∼13 b/min) in Tibetans during treadmill running in hypoxia compared to their Han Chinese counterparts (Kayser et al., 2019). Furthermore, iloprost inhalation improved hypoxic aerobic capacity in Han Chinese but not Tibetans, alluding to hypoxic pulmonary vasoconstriction and right ventricular function as limiting factors of hypoxic performance in Han. In the present study, we observed no between-group difference in SpO_2_ or maximal HR during hypoxic cycling exercise (Figure 2). Given that maximal HR is typically higher during incremental treadmill running compared to incremental cycling in trained and untrained individuals, and is influenced by training mode and postural position [see Millet et al. (2009) for review], we attribute the discrepant HR and SpO_2_ findings to the differences in exercise modality (cycling vs. treadmill running) and postural position (reclined and tilted vs. upright).

### Methodological Considerations

There are several methodological considerations to be taken into account when interpreting our data. First, the PaO_2_ values we obtained in normoxic conditions were higher than expected (∼130 mmHg, Table 2). There are two possible explanations for this. Firstly, the simulated normoxic condition was mildly hyperbaric due to instrumental/chamber setting error. However, independent measure of chamber pressure indicates this was unlikely the case. Alternatively, there was a leak from the O_2_ masks used for the 100% O_2_ breathing which resulted in FIO_2_ being ∼0.22–0.24. This is the most likely explanation since we also observed slightly elevated PaO_2_ in the hypobaric hypoxic condition (Table 2). Accordingly, our findings should be interpreted as mild hyperoxia rather than normoxia. While the cause of the high PaO_2_ values is perplexing, we believe this does not affect our between-group comparisons since both Han Chinese and Tibetans exhibited high PaO_2_ (Table 2).

Second, muscle oximetry based on NIRS signals provides non-invasive and region-specific information of changes in [O_2_Hb] and [HHb] of the skeletal muscle tissue at rest and during exercise [see (Grassi and Quaresima, 2016; Perrey and Ferrari, 2017) for reviews], but there is much debate concerning the relative contributions of Hb and Mb to themuscle NIRS signals. At rest, Mb may contribute as much as ∼80% of the NIRS signal (Tran et al., 1999; Marcinek et al., 2007; Bendahan et al., 2017), but this value varies in relation to blood flow (as during exercise) and ambient PO_2_ (i.e., hypoxia) Spires et al. (2011). Given that Mb plays a crucial role in hypoxic-tolerance in deep-diving mammals (Fago and Jensen, 2015), and is expressed in greater proportions in Tibetans (Gelfi et al., 2004), it is possible the accentuated muscle tissue deoxygenation in Tibetans is the result of greater Mb deoxygenation during hypoxic exercise.

Finally, we recruited a small sample (*n* = 10 in each group) of recreationally active individuals. Tibetans in this study consistently outperformed Han Chinese by ∼32% under both normoxic and hypoxic conditions, suggesting better efficiency during cycling in Tibetans. Wang et al. (2010) found hypoxic training does not affect muscle [O_2_Hb] response to hypoxic exercise nor cerebral NIRS parameters during normoxic exercise, despite a ∼50% improvement in maximal workload (+22 ml.min^–1^.kg V̇O_2_max) in their participants. Recently, Caen et al. (2019) showed aerobic training increased the amplitude of muscle totHb and HHb responses during incremental exercise in normoxia, indicating improved O_2_ availability and muscle O_2_ extraction with improved fitness. Meanwhile, we found comparable muscle tissue oxygenation responses between Han Chinese and Tibetans during incremental exercise in normoxia, and lower muscle O_2_Hb and totHb in Tibetans during hypoxic exercise. We contend that the between-group differences in muscle tissue oxygenation during incremental exercise is due to ethnicity rather than fitness.

## Conclusion

We found distinct differences between Tibetans and Han Chinese in muscle and brain tissue oxygenation changes during exercise. We found Tibetans exhibited a blunted cerebrovascular response to hypocapnia during normoxic exercise. This combined with a higher heart rate (and cardiac output) enabled them to maintain a higher cerebral tissue oxygenation at maximal effort compared to Han Chinese. During hypoxic exercise, we found evidence of greater muscle tissue deoxygenation in Tibetans for a given workload, which we interpret as enhanced muscle O_2_ extraction. Tibetans consistently outperformed their Han Chinese counterpart in both normoxic and hypoxic conditions, exhibiting better energy economy at exercise exertion. For the first time, our data demonstrate that Tibetans can maintain higher cerebral tissue oxygenation during maximal normoxic exercise and enhance muscle O_2_ utilization during hypoxic exercise. Whether these muscular and cerebrovascular responses account for their superior aerobic performance warrants further investigation.

## Data Availability Statement

The raw data supporting the conclusions of this article will be made available by the authors, without undue reservation.

## Ethics Statement

The studies involving human participants were reviewed and approved by the University of Oregon Institutional Review Board and the Qinghai High Altitude Medical Science Institutional Committee. The patients/participants provided their written informed consent to participate in this study.

## Author Contributions

J-LF, TW, AL, and BK contributed to the conception and design of the study. J-LF, TW, LN, WLB, AL, and BK performed the data collection. J-LF, AL, and BK carried out the analysis, carried out the interpretation of the data, and contributed to the revision of the manuscript. J-LF drafted the manuscript and prepared the figures. All authors approved the final version of the manuscript.

## Conflict of Interest

The authors declare that the research was conducted in the absence of any commercial or financial relationships that could be construed as a potential conflict of interest.
